# The lncRNA ENSG00000254041.1 promotes cell invasiveness and associates with poor prognosis of pancreatic ductal adenocarcinoma

**DOI:** 10.18632/aging.102835

**Published:** 2020-02-23

**Authors:** Bo Chen, Qiqi Zhang, Xujing Wang, Yongkun Wang, Jiaqu Cui, Huiren Zhuang, Jianying Tang

**Affiliations:** 1 Department of Hepatopancreatobiliary Surgery, East Hospital Affiliated to Tongji University, Shanghai 200123, China

**Keywords:** pancreatic cancer, lncRNA, invasion, chemoresistance

## Abstract

Pancreatic cancer (PC) mainly occurs after 60 years of age, and its prognosis remains poor despite modest improvements in recent decades. Long non-coding RNAs (lncRNAs) are well known as a class of transcripts involved in cancer occurrence and progression. The process of epithelial to a mesenchymal (EMT) phenotype in tumor cell increases their migratory and invasive properties, resulting in facilitating metastasis. Here, we reanalyzed RNA-seq data from the TCGA PC database and identified that ENSG00000254041.1 increasingly expressed in samples with elevated EMT signature score. Then, the evaluated expression and prognostic significance of ENSG00000254041.1 were verified in our cohort. Meanwhile, multivariate analysis suggested that ENSG00000254041.1 was independent factors for predicting the prognosis of PC, apart from advanced stage (III/IV). Moreover, functional assay revealed that knock down of ENSG00000254041.1 significantly decreased proliferation, invasion and chemoresistance of PC cells (SW1990 and BxPC-3), while overexpression of ENSG00000254041.1 in PC cells (Panc-1) resulted in the opposite effects. Western blot showed that knockdown of ENSG00000254041.1 expression in PC cells caused a significant downregulation of vimentin, Snail and SOX4, and upregulation of E-cadherin; also, ENSG00000254041.1 overexpression in PC cells resulted in opposite effects. In conclusion, these findings indicated that ENSG00000254041.1 promotes PC progression, and might provide a potential biomarker for predicting the prognosis of PC.

## INTRODUCTION

Human cancers occur in elderly population at an alarming rate, indicating age is the greatest risk factor for cancer. Pancreatic cancer (PC) is a highly lethal malignancy mostly affecting elderly people. The incidence of PC increases with age; in the United States, only 13% of all patients with PC are diagnosed before 60 years of age [1]. Currently, only 20% of patients have resectable PC, with an 80% relapse rate [2, 3]. The majority of PC patients are unresectable disease due to local invasion or widespread metastasis [4]. However, the mechanisms of tumor invasion and metastasis are not yet clearly defined. Therefore, a series of studies in this area and the provision of potential markers are still needed. Epithelial-mesenchymal transition (EMT) in cancer is a process by which the cancer cells lose their cell-cell adhesion capacity and break through the basement membrane for invasion, during the initiation of metastasis [5]. Specimens from PC patients indicate that EMT status is associated with portal vein invasion and lymph node metastasis [6, 7].

Long non-coding RNAs (lncRNAs) is the non-protein coding transcripts with the length over 200 nucleotides [8]. Multiple studies have shown that lncRNAs may be involved in various process of cancer progression including EMT [9, 10]. For example, the overexpression of *HOTAIR* has been suggested to be associated with poor prognosis in PC and is proposed to increase tumor invasiveness and metastasis [11]. LncRNA NORAD enhances the hypoxia-induced EMT to promote PC metastasis by regulating the expression of the small GTP binding protein RhoA [12]. In the present study, we identified a metastasis related lncRNA ENSG00000254041.1 by reanalyzing the cancer genome atlas (TCGA) PC database. Then, expression status, prognosis analysis and functional experiments were performed to confirm our findings. Thus, the new lncRNA could serve as a useful biomarker for predicting the prognosis of PC.

## RESULTS

### ENSG00000254041.1 expression is associated with elevated Epithelial-to-mesenchymal transition

**Figure 1:**
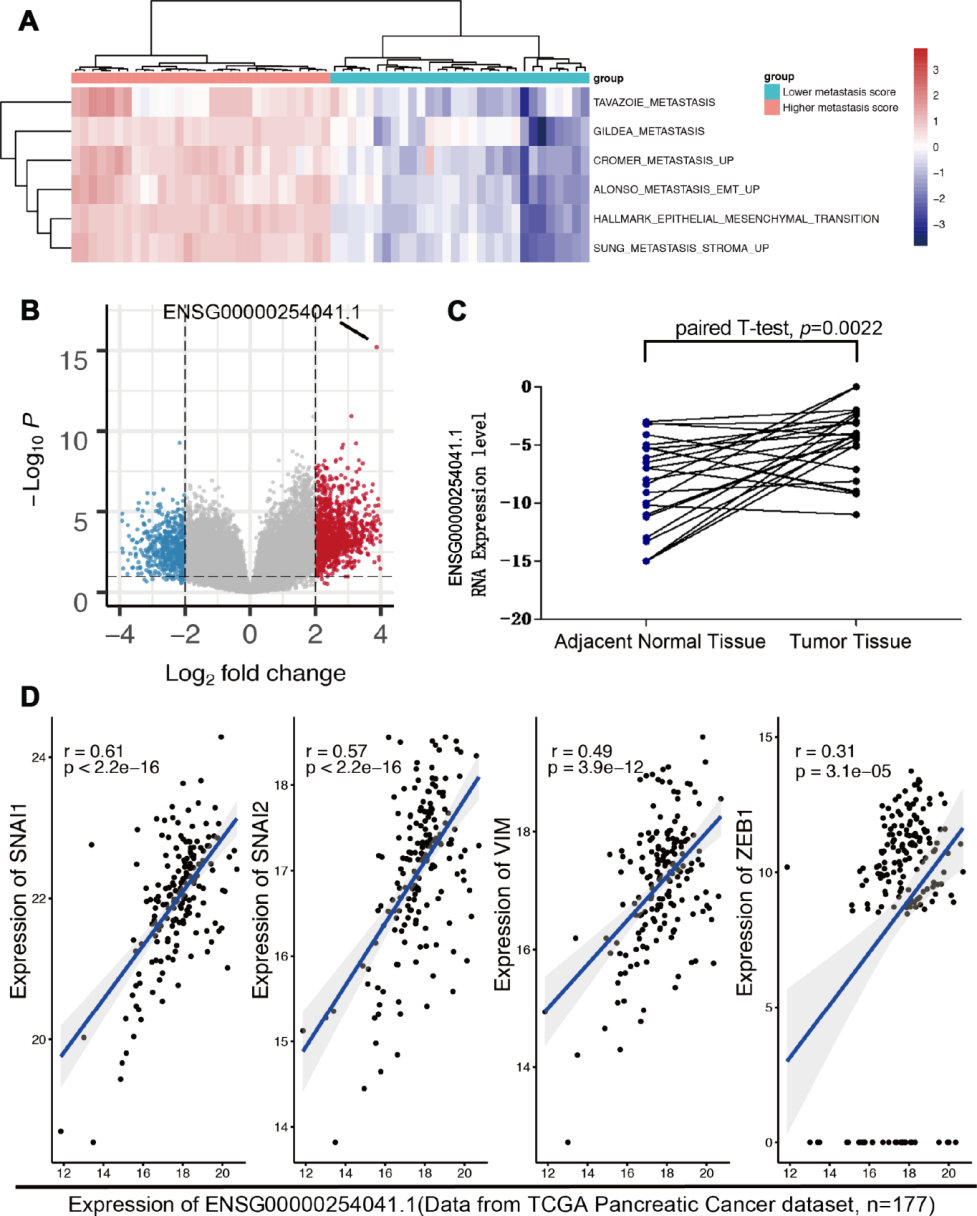
**The expression of ENSG00000254041.1 in PC and adjacent normal tissues.** (**A**) Reanalyzing the RNA-seq data from TCGA PC patients with either high or low epithelial-mesenchymal transition (EMT) signature scores. (**B**) ENSG00000254041.1 was the top-ranked molecule of the different LncRNA between the two groups. (**C**) Expression levels of ENSG00000254041.1 were performed in 24 paired tumor and matched normal tissue samples. (**D**) A positive correlation between ENSG00000254041.1 and individual gene transcripts related to EMT.

The transition of tumor cells with an EMT phenotype increases their migratory and invasive properties, resulting in facilitating metastasis. Here, to elucidate potential targets that inhibit this process, we first characterized PC patients from TCGA with either high or low EMT signature scores. By using ssGSEA gene signature score, each 30 samples bearing highest and lowest ssGSEA score were screened out (Figure 1A). We then performed differential expression analysis and identified that ENSG00000254041.1 was the top-ranked molecule (Figure 1B, Supplementary Table 1). In order to validate the prognostic value of ENSG00000254041.1, we next examined the expression of ENSG00000254041.1 in 24 paired tumors and matched normal tissue samples. We confirmed the expression of ENSG00000254041.1 was significantly increased in PC compared to adjacent normal tissue (Figure 1C). Further analyses revealed a positive correlation between ENSG00000254041.1 and individual gene transcripts related to EMT, including SNAI1, SNAI2, VIM and ZEB1 (Figure 1D).

### Prognosis analysis of ENSG00000254041.1

**Figure 2:**
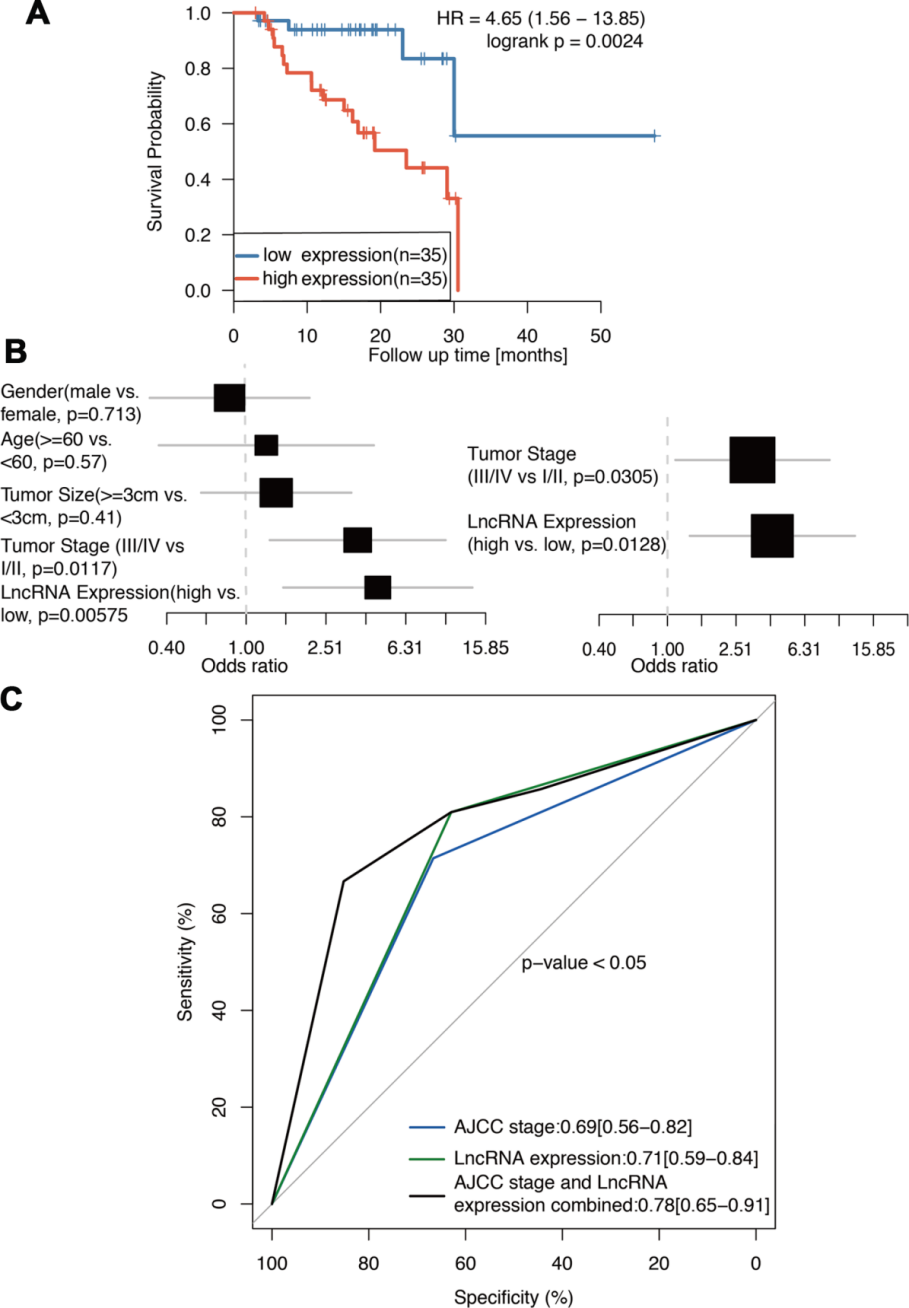
**ENSG00000254041.1 is prognostics biomarker of PC.** (**A**) Survival analysis of 70 PC patients with high and low expression of the ENSG00000254041.1 in our cohort. (**B**) Univariate and multivariate regression analyses (all bars correspond to the 95% confidence intervals). (**C**) ROC analysis of ENSG00000254041.1 expression level for survival prediction of patients with PC.

We further analyzed the association between ENSG00000254041.1 and clinical outcome of patients. The expression level of ENSG00000254141.1 in the 70 tumor tissue samples was measured by RT-PCR and these samples were divided into two groups according to the expression level of ENSG00000254041.1 (median value was used as the cut-off value). Survival analysis showed that high expression group was associated with shorter overall survival than low expression was [20.25 months (95% CI 16.456-24.043) vs. 42.97 months (95% CI 29.477-56.472), *p*=0.0024] (Figure 2A). In the univariate Cox regression analysis, ENSG00000254041.1 expression and clinical stage were found to be predictive for clinical outcome. Moreover, multivariate analysis showed that evaluated ENSG00000254041.1 expression and advanced stage (III/IV) remained the independent predictors for clinical outcome (Figure 2B). ROC curves were determined to evaluate the sensitivity and specificity of the survival prediction based on the lncRNA expression and the AJCC stages (Figure 2C). Interestingly, the AUC for ENSG00000254041.1 and AJCC stage combined could improve the survival prediction (AUC, 0.78 versus 0.71) (Figure 2C).

### ENSG00000254041.1 affects cell cycle and cell proliferation *in vitro*

**Figure 3:**
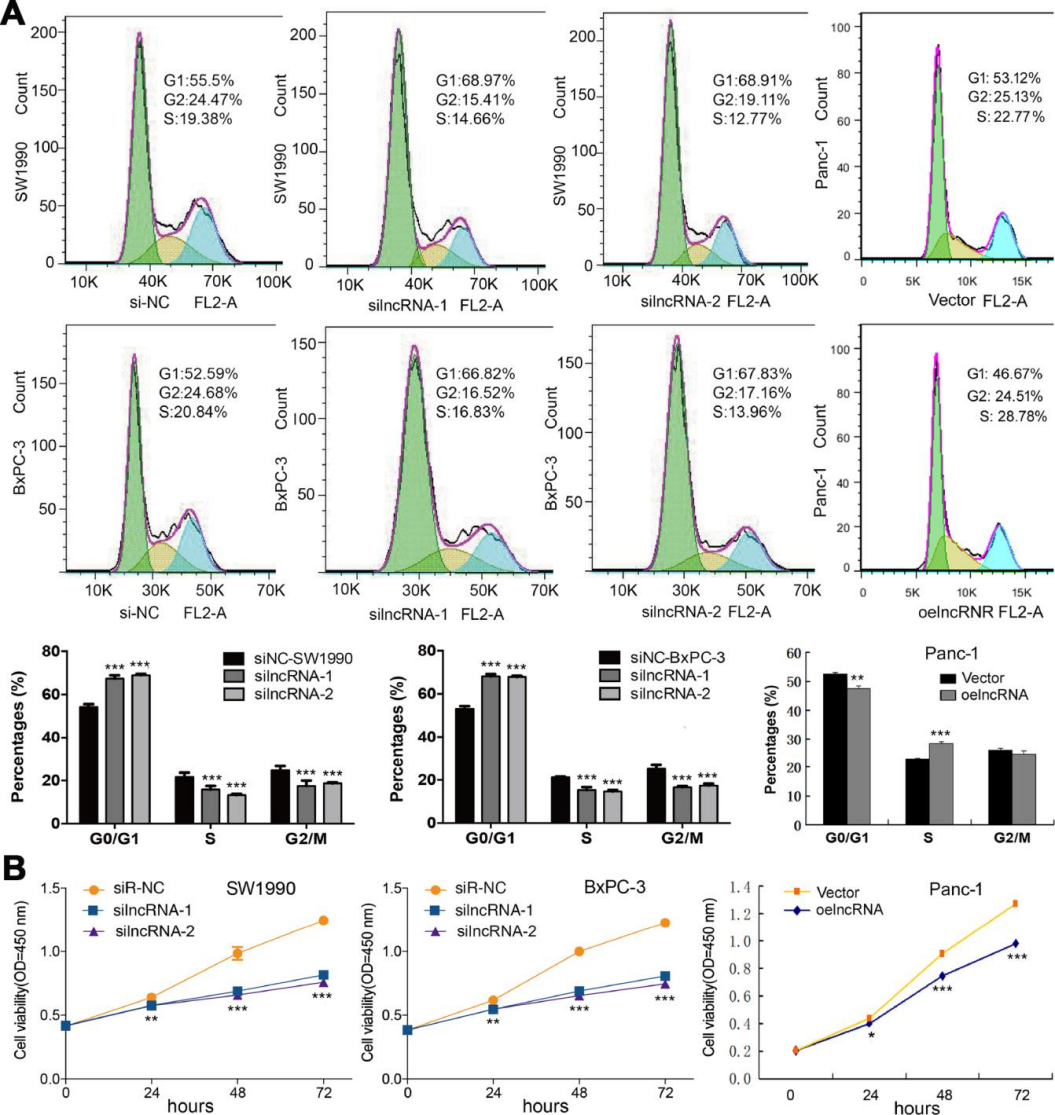
**ENSG00000254041.1 promotes cell cycle and cell proliferation in PC cells.** (**A**) Knockdown of the ENSG00000254041.1 expression decreased the percentage of S-phase and increased G0/G1 phase cell in both of SW1990 and BxPC-3 cells, while overexpression of ENSG00000254041.1 in Panc-1 cells resulted in the opposite effects. (**B**) Downregulation of ENSG00000254041.1 inhibited proliferation of SW1990 and BxPC-3, while overexpression of ENSG00000254041.1 promoted Panc-1 cells proliferation. Three samples in each group. *p < 0.05, **p < 0.01, ***p < 0.001 compared with si-NC or vector.

Based on the baseline expression levels of ENSG00000254041.1 in human PC cell lines, we constructed siRNAs into two PC cells (SW1990 and BxPC-3), and an overexpression vector into Panc-1 cells (Supplementary Figure 1). Three siRNAs were designed to test their suppression efficiency. Among them, siRNAi-1 and siRNAi-2 were the more efficient with a knockdown ratio of >70% in both cell lines. Thus, siRNAi-1, siRNAi-2 or siRNAi-mixure was used for knockdown studies. Knockdown of the ENSG00000254041.1 expression decreased the percentage of S phase and increased the percentage of G_0_/G_1_ phase in both of SW1990 and BxPC-3 cells, while overexpression of ENSG00000254041.1 in Panc-1 cells resulted in the opposite effects of cell cycle (Figure 3A). A cell proliferation assay also showed that the downregulation of ENSG00000254041.1 inhibited PC cells proliferation, while overexpression of ENSG00000254041.1 promoted Panc-1 cells proliferation (Figure 3B).

### ENSG00000254041.1 drives Epithelial-Mesenchymal Transition (EMT) by controlling SOX4 expression

**Figure 4:**
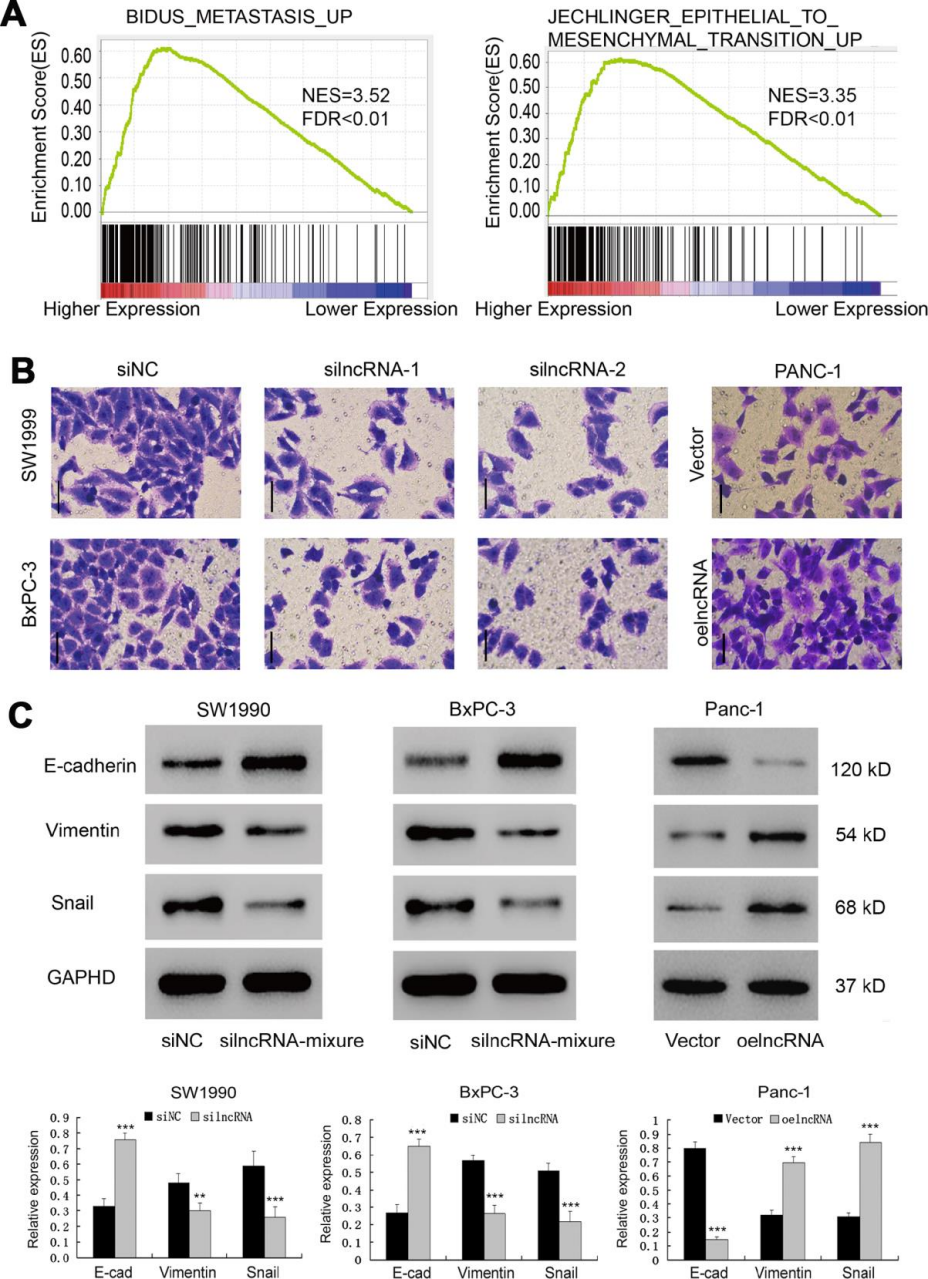
**ENSG00000254041.1 drives Epithelial-Mesenchymal Transition (EMT).** (**A**) GSEA analysis revealed that ENSG00000254041.1 expression associated with the EMT pathway. (**B**) Decreased expression of ENSG00000254041.1 in SW1990 and BxPC-3 cells significantly reduced their invasive capacity, while overexpression of ENSG00000254041.1 promoted Panc-1 cells invasion, as measured by a transwell assay. (**C**) Western blot analysis confirmed that a set of EMT-related proteins was affected by ENSG00000254041.1. Three samples in each group. **p < 0.01, ***p < 0.001 compared with si-NC or vector.

To investigate the ENSG00000254041.1-associated pathways in an unbiased manner, we performed GSEA using the GSE15471 dataset. The EMT pathway had the strongest association with ENSG00000254041.1 expression in the multi-tumor dataset (Figure 4A, Supplementary Figure 2). In order to determine whether ENSG00000254041.1 could influence the metastatic capacity of PC, a transwell assay was performed. The decreased expression of ENSG00000254041.1 significantly inhibited cell invasion capability in SW1990 and BxPC-3 cells, while overexpression of ENSG00000254041.1 promoted Panc-1 cells invasion (Figure 4B). Furthermore, western blot showed that knockdown of ENSG00000254041.1 in SW1990 and BxPC-3 cells caused a significant downregulation of mesenchymal markers (vimentin and Snail) and upregulation of epithelial marker (E-cadherin); while ENSG00000254041.1 overexpression in Panc-1 cells could increase the expression of vimentin and Snail, and decrease E-cadherin expression (Figure 4C). These results indicated that ENSG00000254041.1 may driver EMT in PC.

**Figure 5:**
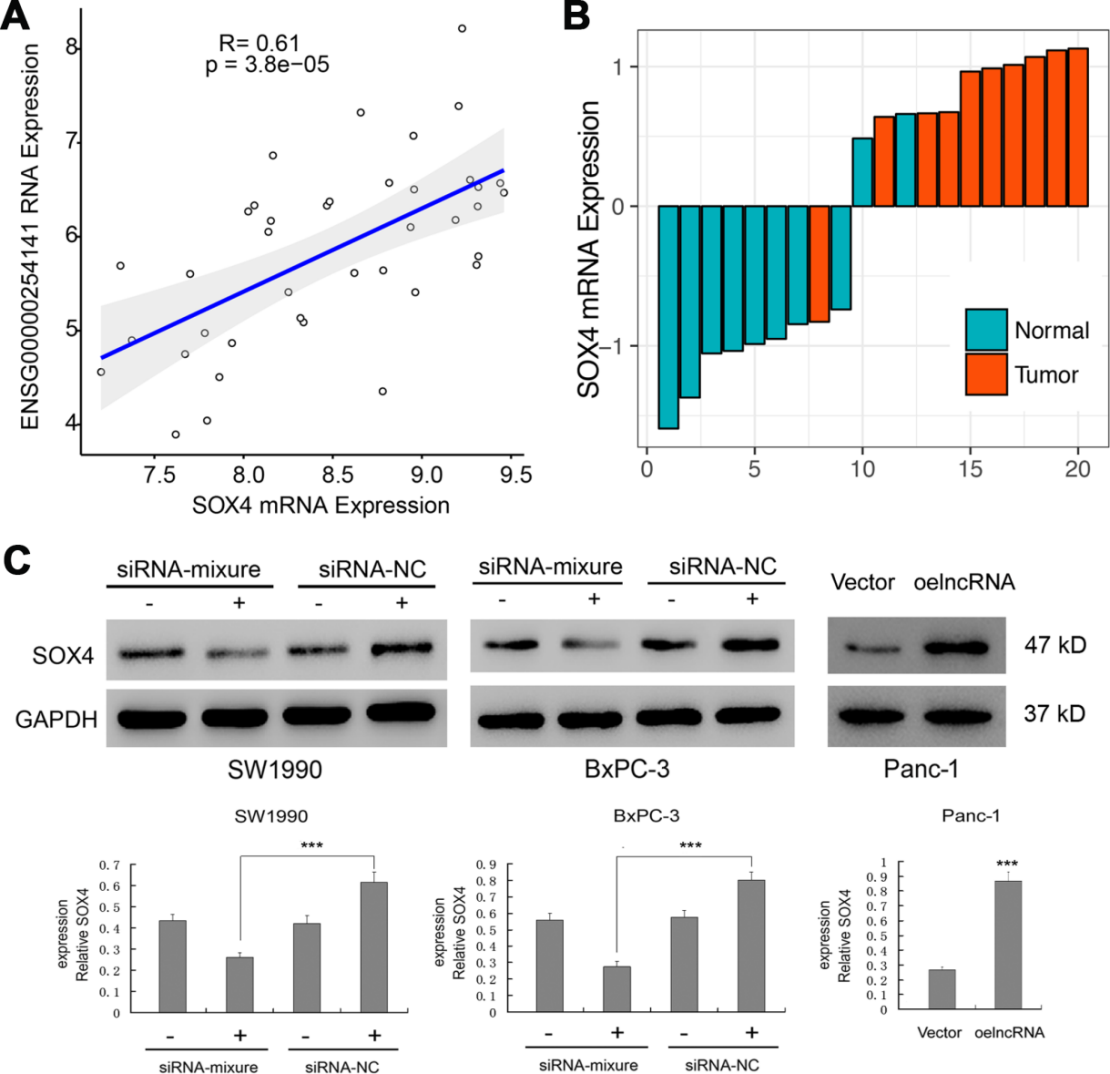
**Regulation of the SOX4 expression by ENSG00000254041.1.** (**A**) Correlation analysis between the expression of ENSG00000254041.1 and SOX4 in the “Liao Metastasis” pathway. (**B**) SOX4 expression levels were examined in 10 paired PC tissues and their adjacent non-neoplastic tissues by RT-PCR. (**C**) ENSG00000254041.1 affected the expression of SOX4 protein in the PC cell lines. Three samples in each group. ***p < 0.001 compared with vector or indicated groups.

Among the significantly affected pathways, "Liao Metastasis" was assigned with the highest enrichment score. By analyzing the correlation between ENSG00000254041.1 and mRNAs in the Liao Metastasis pathway, we found SOX4 with the highest correlation coefficient in this pathway (Pearson correlation, R=0.61; *p*=3.8e-05; Figure 5A). We used RT-PCR to measure the expression of SOX4 in 10 tumor tissues and 10 normal tissues. We observed enhanced expression of SOX4 levels in tumor tissues of PC samples (Figure 5B). Thus, we hypothesized that ENSG00000254041.1 may involve in the EMT process of PC via regulation of the SOX4 expression. As expected, knockdown of ENSG00000254041.1 inhibited the expression of SOX4 in SW1990 and BxPC-3 cells, and overexpression of ENSG00000254041.1 in Panc-1 cells increased the expression of SOX4 (Figure 5C).

### ENSG00000254041.1 knockdown enhances the chemosensitivity of human PC cells to gemcitabine *in vitro*

**Figure 6:**
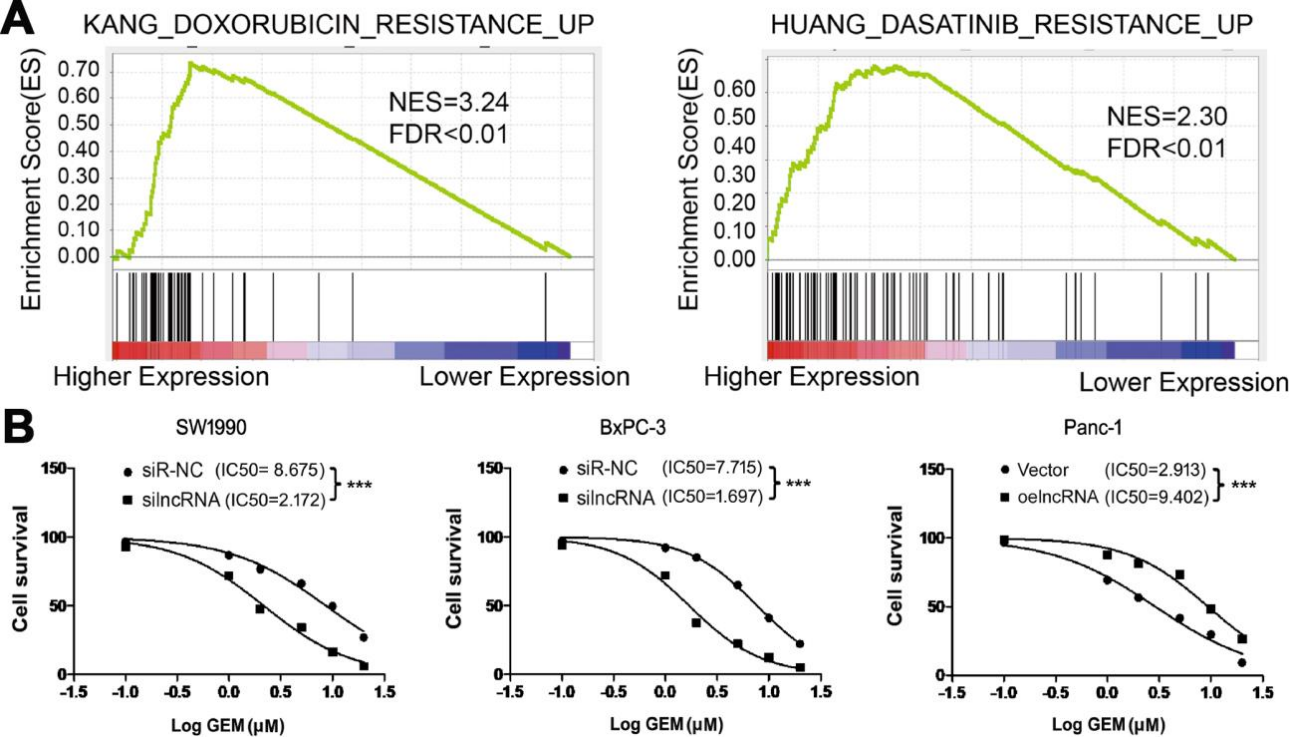
**ENSG00000254041.1 increase the chemoresistance of PC cells to gemcitabine *in vitro*.** (**A**) GSEA analysis revealed that ENSG00000254041.1 expression associated with the multi-drug resistance. (**B**) Knockdown of ENSG00000254041.1 decreased the IC_50_ values of GEM in SW1990 and BxPC-3 cells, and overexpression of ENSG00000254041.1 in Panc-1 cells increased the IC_50_ values of GEM. ***p < 0.001 compared with indicated group.

Innate or acquired resistance to chemotherapy is a hallmark of PC. We next performed GSEA to investigate whether ENSG00000254041.1 expression contributes to the chemoresistance of PC patients. We found that ENSG00000254041.1 expression is associated with multiple chemoresistance pathways in PC patients (Figure 6A). Cell viability assay further verified that knockdown of ENSG00000254041.1 decreased the IC_50_ values of gemcitabine (GEM) in SW1990 (from 8.675 to 2.172 μM, *p*<0.001) and BxPC-3 cells (from 7.715 to 1.697 μM, *p*<0.001), and overexpression of ENSG00000254041.1 in Panc-1 cells increased the IC_50_ values of GEM (from 2.913 to 9.402 μM, *p*<0.001) (Figure 6B).

## DISCUSSION

Accumulated evidence has revealed that lncRNAs are involved in the pathogenesis of different types of cancers including PC [13, 14], which may serve as potential biomarkers for the early detection and prognostic predictions of PC [15]. In the present study, we identified a metastasis related lncRNA by reanalyzing the cancer genome atlas (TCGA) PC database. Then, the evaluated expression and prognostic significance of ENSG00000254041.1 were verified in our cohort. Functional assay revealed that ENSG00000254041.1 enhanced the proliferation, migration, invasion and chemoresistance of PC cells. ENSG00000254041.1 is located on chromosome 8q23.1, which has a transcript length of 716 nucleotides. No study reveals an oncogenic role for ENSG00000254041.1 in cancer. These findings indicated that ENSG00000254041.1 is a new lncRNA play oncogenic roles in the progression of PC.

EMT, a process that epithelial cells lose the apical-basal polarity and cell–cell adhesion, and transit to invasive mesenchymal cells, is involved in numerous biological and pathological processes, including embryonic development, wound healing, cancer cell metastasis and drug resistance [16, 17]. Cells undergoing EMT display decreased expression level of epithelial genes (such as E-cadherin, ZO-1 and occludin) and increased expression level of mesenchymal genes (such as N- cadherin, vimentin and fibronectin). The changes in gene expression during EMT lead to numerous phenotypic changes, such as cell morphological changes, loss of adhesion, gain of stem cell-like features, and chemotherapy resistance [18–20]. The EMT phenotype had the strongest association with ENSG00000254041.1 expression in the multi-tumor dataset by GSEA analysis, and was verified by western blot in our study.

SOX4 is a member of the Sox (SRY-related HMG-box) family of transcription factors with a critical role in embryonic development [21]. Evaluated SOX4 gene expression is observed in many cancer types, and increased SOX4 activity contributes to cellular transformation and metastasis [22, 23]. Recent report documents that SOX4 plays a central role in EMT as well as in primary tumor growth and metastasis by directly regulating the expression of a number of genes with critical functions in EMT [24]. Previous study has found that SOX4 expression is upregulated by a lncRNA CASC15 and promotes cell proliferation in acute leukemia [25]. Herein we revealed that ENSG00000254041.1 may promote the EMT process of PC via regulation of the SOX4 expression.

It should be acknowledged that there are some limitations in this study. Although the Affymetrix Human Genome U133 Plus 2.0 array has a large number of probe sets, all the lncRNAs are not covered by the array. Thus, part of lncRNAs correlated with biological behavior of PC might not be identified [26]. In addition, the ethnic group of our cohort was Chinese; these results should be interpreted with great caution while they were applied to patients of other population. Finally, our knowledge of biological functional of most lncRNAs is still limited. More experimental investigations are needed to better understand their function in cancer pathogenesis. Meanwhile, further clinical studies are necessary to the clinical utility of these lncRNAs in pancreatic cancer prognosis evaluation.

In summary, we identified a new lncRNA ENSG00000254041.1 is significantly upregulated in PC, thus promoting PC progression, and might provide a potential biomarker for predicting the prognosis of PC.

## MATERIALS AND METHODS

### TCGA data collection and analysis

Gene expression data of PC samples from TCGA were collected from the FireHose data repository (https://gdac.broadinstitute.org/). Clinical data were also retrieved from the same source. The metastasis gene expression signature was retrieved from MSigDB hallmark gene sets and C2 curated gene sets (http://software.broadinstitute.org/gsea/msigdb/index.jsp). We used the single-sample Gene Set Enrichment analysis algorithm, implemented in R package GSVA [27], to calculate a metastasis signature score for each sample. Default parameters from the GSVA package were used. The voom function is used to for differential expression (DE) analysis of RNA-seq data. Voom is a function in the limma package that modifies RNA-Seq data for use with limma. Correlation analysis was used to quantify the association between LncRNA and EMT related gene.

### Patients and tissue collections

In total, the primary tumor samples were collected from 70 patients at East Hospital Affiliated to Tongji University who underwent surgery for PC between 2013 and 2014. Tumor tissues and corresponding normal tissues (>2 cm from tumor) were stored in liquid nitrogen for future use. None of these patients have received preoperative radiotherapy or chemotherapy. All the PC tissues were double-checked by experienced pathologists. Tumor stage was assessed based on the 2017 tumor node metastasis (TNM) classification of malignant tumors by the American Joint Committee on Cancer (AJCC). Regular follow-up was carried out through phone calls or clinic visits every 3 months until May 2018 or the date of death. This study protocol was approved by the Ethics Committee of East Hospital Affiliated to Tongji University and written informed consents were provided prior to enrollment of patients.

### RNA extraction and reverse transcription quantitative PCR analysis

We extracted total RNA from target cells by using Trizol reagent (Invitrogen, Carlsbad, CA, USA). Then Reverse Transcription System Kit (Takara, Dalian, China) was used to synthesize the first-strand from 1 μg of total RNA. The RNA levels were measured using a real-time quantitative PCR system (SYBR Premix Ex Taq kit, Takara, Dalian, China, ABI 7500 Real-Time PCR System, Applied Biosystems, CA, USA). GAPDH was used as an endogenous control gene, and each assay was performed in triplicate. The primers used for PCR of ENSG00000254041.1 were as follows: forward: 5′-GGAAATTCTGCTTATCATCGG-3′, reverse: 5′-AAGTGCCTGAGAGGCCAAAA-3′.

### Cell culture and transfection

Human PC cell lines (SW1990, BxPC-3 and) were obtained from the Shanghai Cell Bank (Shanghai, China). The SW1990, BxPC-3 and Panc-1 cell lines were cultured in Dulbecco's modied Eagle's medium (DMEM) or RPMI-1640 supplemented with 10% fetal bovine serum (FBS), 100 U/ml penicillin and 0.1 mg/ml streptomycin, in an incubator at 37°C with 5% CO_2_ atmosphere. Small interference RNA (siRNA) for the knockdown of lncRNA and negative control siRNA were constructed by GenePharma (Shanghai, China). Transfection was performed using Lipofectamine 2000 (Invitrogen, Carlsbad, CA, USA) according to the manufacturer’s instructions. The primers of siRNA target used in this study were as follows: siRNA-1: 5′-TGGTGGAGCCTAAAGACCTGAGGAT-3′; siRNA-2:5′GCTGCAGCAGAACACGAATATCAAT-3′; siRNA-3: 50027-GAGCCCGGCTCACATAATCAGCAAA-3′; si-NC: 5'-TGGGAGTCCAAACAGGTCGATGGA-3′. Complementary DNA encoding ENSG00000254041.1 was synthesized and subcloned into the pcDNA3.1(+) vector (Invitrogen) according to the manufacturer’s instructions. Transfections were performed using Lipofectamine 3000 and OPTI-MEM (Invitrogen) according to the manufacturer’s instructions. Cells transfected with scramble siRNA (siNC) or infected with blank pCDNA3.1(+) vector (blank vector) were used as negative controls.

### Cell viability and proliferation

Cell proliferation was assessed using cell counting assay Kit-8 (CCK-8) according to the manufacture’s protocol. Subsequent to the plating of cells in 96-well plates, the addition of CCK-8 reagents was made to every well. Proliferation rates were determined at each time point. The absorbance was measured at a wave length of 490 nm by a microplate reader (ELx800; Bio-Tek, Winooski, VT, USA). Using GraphPad Prism 5.0 (GraphPad Software, La Jolla, CA, USA), IC_50_ values were calculated by a GEM concentration-response curve (concentration gradient: 0.1, 1, 2, 5, 10 and 20 μM for a 48-h treatment period).

### Cell migration and invasion assay

PC cells were grown to confluence in 24-well transwell chambers (8 mm pore size; BD Biosciences, San Jose, CA, USA) and were treated with siRNA or control siRNA. Matrigel-coated invasion and stained using 0.1% crystal violet. Numbers of invaded cells were counted in five randomly selected fields under microscope.

### Western blotting analysis

The whole cell extracts were prepared using RIPA buffer (Beyotime, Shanghai, China). After electrophoresis, proteins were electroeluted at 120 volts onto a polyvinylidene difluoride (PVDF) membrane. The membrane was incubated with primary antibodies against rabbit anti-human E-cadherin (1:500, #ab40772, Abcam), rabbit anti-human Vimentin (1:500; #ab92547, Abcam), rabbit anti-human Snai1 (1:1000, #ab180714, Abcam), and GAPDH (1:2000, CST) overnight at 4°C and then incubated with a secondary antibody. GAPDH was used as a control for total protein input.

### Gene set enrichment analysis

Gene set enrichment analysis (GSEA) algorithm was used to identify the pathways that were significantly enriched between ENSG00000254041.1 low and high tumor samples. A ranked gene list was generated by comparing the mRNA microarray data of ENSG00000254041.1 low group with ENSG00000254041.1 high group (ENSG00000254041.1 low vs. high). Then enrichment of different pathway gene sets in this ranked gene list were evaluated using GSEA.

### Statistical analysis

All statistical analysis was performed using R program (version 3.41). Wilcoxon tests were performed to explore the associations between the lncRNA expression levels. The survival data was estimated using Kaplan Meier method. Univariate and multivariate Cox regression analysis were carried out to analyze the effects of lncRNA expression on patients’ survival. The ROC curve was plotted by pROC package. All *p* values were two sided, and a *p* value < 0.05 was considered to be statistically significant.

## Supplementary Material

Supplementary Figures

Supplementary Table 1
