# Supplementary material for: The lncRNA ENSG00000254041.1 promotes cell invasiveness and associates with poor prognosis of pancreatic ductal adenocarcinoma
Source: Aging (Albany NY). 2020 Feb 23;12(4):3647–61. doi: 10.18632/aging.102835 (PMC7066894; doi:10.18632/aging.102835)
Supplement: Supplementary Figures [file aging-12-102835-s002..pdf]

SUPPLEMENTARY FIGURES

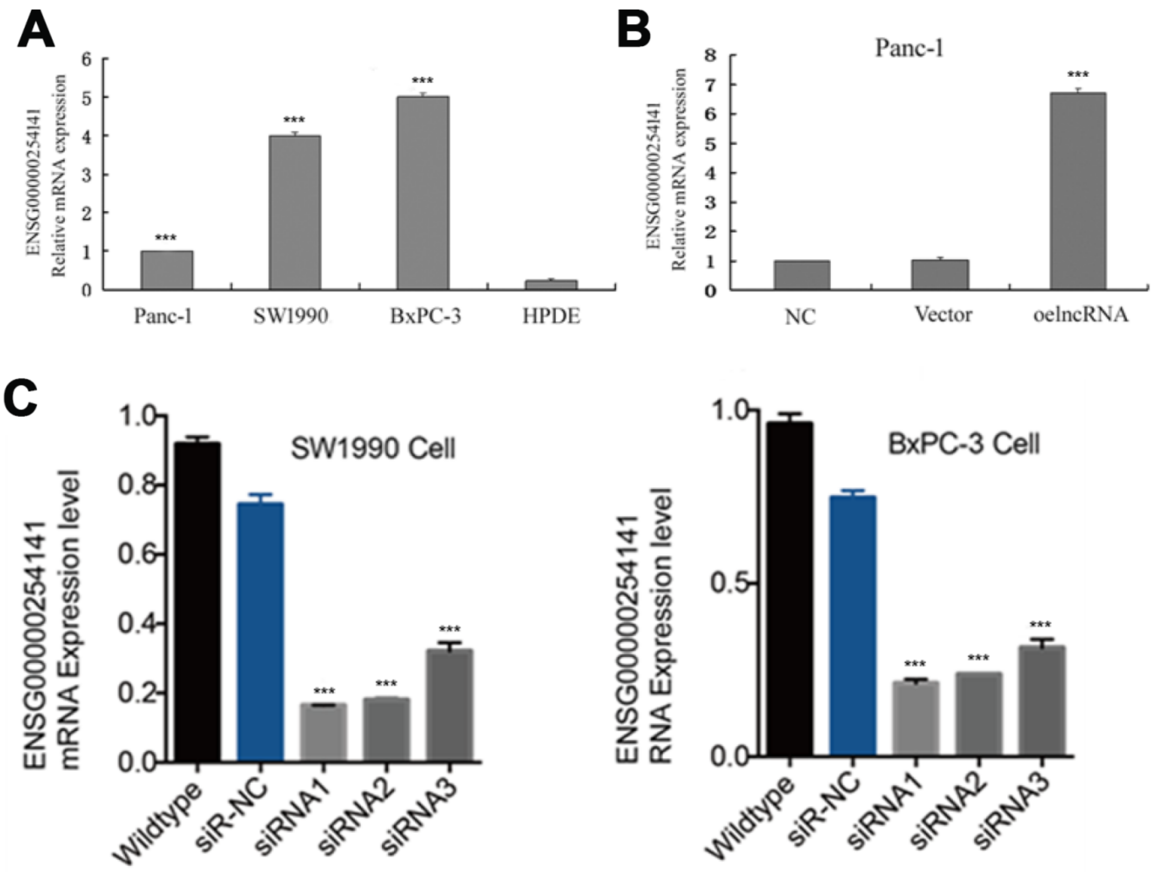

Supplementary Figure 1. (**A**) The baseline expression levels of ENSG00000254041.1 in human pancreatic cancer cell lines (SW1990, BxPC-3 and Panc-1) and normal pancreatic epithelial cell line (HPDE), ***p < 0.001 compared with HPDE. (**B**) The transfection efficiency of overexpression vector in Panc-1 cell line. ***p < 0.001 compared with NC and Mock vector. (**C**) The suppression efficiency of three siRNAs in SW1990 and BxPC-3 cell lines. ***p < 0.001 compared with si-NC.

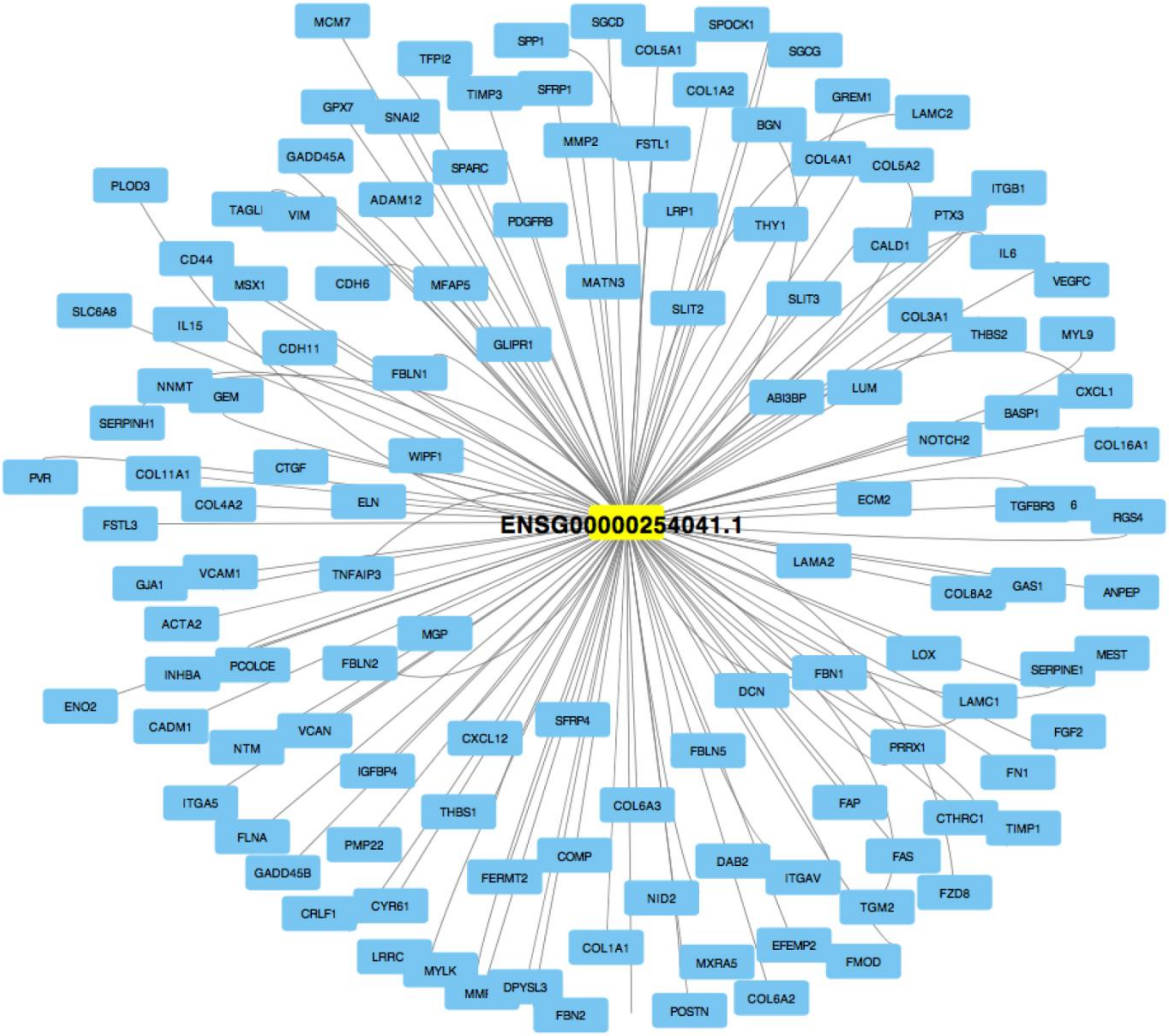

Supplementary Figure 2. LncRNA ENSG00000254041 and EMT Pathway Co-expression Analysis.
