# Supplementary material for: The lncRNA ENSG00000254041.1 promotes cell invasiveness and associates with poor prognosis of pancreatic ductal adenocarcinoma
Source: Aging (Albany NY). 2020 Feb 23;12(4):3647–61. doi: 10.18632/aging.102835 (PMC7066894; doi:10.18632/aging.102835)
Supplement: Supplementary Table 1 [file aging-12-102835-s001..docx]

**Supplementary Table 1.** Differentially expressed genes between the samples with high or low epithelial to a mesenchymal signature scores

| ENSG_ID | logFC | t-statistics | P.Value |
| --- | --- | --- | --- |
| **ENSG00000254041.1** | **3.87674291** | **25.8615386** | **6.07E-16** |
| ENSG00000075884.11 | 1.93654242 | 14.7175437 | 1.23E-11 |
| ENSG00000250969.1 | 3.10245599 | 14.7734749 | 1.15E-11 |
| ENSG00000054803.3 | 4.77454483 | 12.6801136 | 1.51E-10 |
| ENSG00000184347.13 | 3.24596979 | 11.6857864 | 5.82E-10 |
| ENSG00000185761.9 | -2.1695182 | -11.743524 | 5.37E-10 |
| ENSG00000113088.5 | 2.80964336 | 11.240423 | 1.09E-09 |
| ENSG00000255124.1 | 2.8531985 | 11.5883887 | 6.67E-10 |
| ENSG00000141540.9 | 1.30436306 | 10.9043111 | 1.79E-09 |
| ENSG00000173269.12 | 1.43762834 | 10.6058475 | 2.79E-09 |
| ENSG00000122679.7 | 1.58746567 | 10.4086727 | 3.76E-09 |
| ENSG00000111341.8 | 2.77046746 | 10.2081086 | 5.12E-09 |
| ENSG00000137033.10 | 3.32648054 | 10.0599182 | 6.44E-09 |
| ENSG00000104324.14 | 0.87384985 | 10.0433995 | 6.61E-09 |
| ENSG00000163792.6 | 4.44531393 | 10.4851073 | 3.35E-09 |
| ENSG00000163564.13 | 2.27672216 | 10.028709 | 6.76E-09 |
| ENSG00000180828.2 | 2.62290828 | 9.94125322 | 7.76E-09 |
| ENSG00000187955.10 | 3.44533134 | 9.78898816 | 9.88E-09 |
| ENSG00000139155.7 | 2.62982238 | 10.0790503 | 6.25E-09 |
| ENSG00000117643.13 | 1.51455375 | 9.63386535 | 1.27E-08 |
| ENSG00000117091.8 | 2.23036795 | 9.6259042 | 1.28E-08 |
| ENSG00000139193.3 | 2.09832261 | 9.60174482 | 1.33E-08 |
| ENSG00000106809.9 | 4.20939257 | 9.51272945 | 1.54E-08 |
| ENSG00000170989.8 | 1.81289245 | 9.4734178 | 1.64E-08 |
| ENSG00000211647.1 | 4.33515334 | 9.87981328 | 8.55E-09 |
| ENSG00000131477.9 | 1.61979199 | 9.42763107 | 1.77E-08 |
| ENSG00000119699.6 | 2.31172287 | 9.40159077 | 1.85E-08 |
| ENSG00000110448.9 | 2.00775555 | 9.38036122 | 1.91E-08 |
| ENSG00000183722.7 | 2.06130016 | 9.3947862 | 1.87E-08 |
| ENSG00000140092.13 | 2.34948875 | 9.35705016 | 1.99E-08 |
| ENSG00000183918.13 | 2.32597203 | 9.37862348 | 1.92E-08 |
| ENSG00000150048.9 | 1.42812631 | 9.3209202 | 2.11E-08 |
| ENSG00000141338.12 | 3.34526046 | 9.26832257 | 2.30E-08 |
| ENSG00000154721.13 | 1.76993106 | 9.25479741 | 2.35E-08 |
| ENSG00000204136.9 | 2.13500017 | 9.14050291 | 2.85E-08 |
| ENSG00000226403.1 | 2.99334404 | 9.3764237 | 1.93E-08 |
| ENSG00000198121.12 | 1.5166211 | 9.04792317 | 3.32E-08 |
| ENSG00000107562.15 | 3.26098397 | 9.06609034 | 3.22E-08 |
| ENSG00000186517.12 | 1.42315882 | 9.04606716 | 3.33E-08 |
| ENSG00000182162.8 | 2.07564711 | 8.89763662 | 4.29E-08 |
| ENSG00000027075.12 | 1.16390481 | 8.83129358 | 4.80E-08 |
| ENSG00000101265.14 | 1.72849227 | 8.83582879 | 4.76E-08 |
| ENSG00000104814.11 | 1.67477875 | 8.79189845 | 5.13E-08 |
| ENSG00000155962.11 | 1.75764778 | 8.7823503 | 5.22E-08 |
| ENSG00000110848.7 | 2.02967682 | 8.77258273 | 5.30E-08 |
| ENSG00000138061.10 | 2.78583883 | 8.77625721 | 5.27E-08 |
| ENSG00000144649.7 | 2.05789819 | 8.73448931 | 5.66E-08 |
| ENSG00000139187.8 | 1.63571047 | 8.76588272 | 5.37E-08 |
| ENSG00000169744.11 | 1.34876329 | 8.69495606 | 6.06E-08 |
| ENSG00000179144.4 | 2.23269649 | 8.6848172 | 6.17E-08 |
| ENSG00000174175.15 | 2.45102137 | 8.68463356 | 6.17E-08 |
| ENSG00000198771.9 | 1.77045112 | 8.65483795 | 6.50E-08 |
| ENSG00000155849.14 | 1.48732367 | 8.59995101 | 7.15E-08 |
| ENSG00000180769.7 | 1.30829422 | 8.63132894 | 6.77E-08 |
| ENSG00000268027.4 | 2.21388292 | 8.68925758 | 6.12E-08 |
| ENSG00000000971.14 | 1.55247242 | 8.64669131 | 6.59E-08 |
| ENSG00000164946.18 | 2.63329347 | 8.55016226 | 7.79E-08 |
| ENSG00000221818.7 | 2.90864262 | 8.54727247 | 7.83E-08 |
| ENSG00000100626.15 | 2.27940088 | 8.53238981 | 8.04E-08 |
| ENSG00000181804.13 | 1.87637855 | 8.52222216 | 8.18E-08 |
| ENSG00000017427.14 | 4.83987963 | 8.56599161 | 7.58E-08 |
| ENSG00000187513.8 | 1.47201837 | 8.51398792 | 8.30E-08 |
| ENSG00000124203.5 | 2.65424013 | 8.49837262 | 8.53E-08 |
| ENSG00000198851.8 | 1.78830813 | 8.45002041 | 9.28E-08 |
| ENSG00000163519.12 | 2.1912046 | 8.52370147 | 8.16E-08 |
| ENSG00000129596.4 | 1.78666413 | 8.43349846 | 9.56E-08 |
| ENSG00000112936.17 | 4.55221625 | 8.5254971 | 8.14E-08 |
| ENSG00000120156.19 | 1.83697124 | 8.40994345 | 9.96E-08 |
| ENSG00000136160.13 | 2.43506224 | 8.39718102 | 1.02E-07 |
| ENSG00000133574.8 | 1.65434265 | 8.39797755 | 1.02E-07 |
| ENSG00000186265.8 | 2.50822811 | 8.46215925 | 9.09E-08 |
| ENSG00000157554.17 | 1.81202904 | 8.34883221 | 1.11E-07 |
| ENSG00000012211.11 | -1.1720248 | -8.3368081 | 1.13E-07 |
| ENSG00000280339.1 | 2.24921214 | 8.36061922 | 1.09E-07 |
| ENSG00000106511.5 | 2.28791127 | 8.30149686 | 1.21E-07 |
| ENSG00000073861.2 | 1.66102741 | 8.35609421 | 1.10E-07 |
| ENSG00000261371.4 | 1.5329016 | 8.38370304 | 1.04E-07 |
| ENSG00000120833.12 | 1.69598723 | 8.27147127 | 1.27E-07 |
| ENSG00000091972.17 | 1.79836305 | 8.26334318 | 1.29E-07 |
| ENSG00000117090.13 | 1.75707247 | 8.21445594 | 1.41E-07 |
| ENSG00000181896.10 | 0.69549167 | 8.19669723 | 1.45E-07 |
| ENSG00000267272.4 | 1.65988657 | 8.23316738 | 1.36E-07 |
| ENSG00000178562.16 | 2.07166691 | 8.19795564 | 1.45E-07 |
| ENSG00000196616.11 | 5.24104932 | 8.19776718 | 1.45E-07 |
| ENSG00000174059.15 | 1.694658 | 8.23480391 | 1.36E-07 |
| ENSG00000137509.9 | 1.35010841 | 8.24831185 | 1.33E-07 |
| ENSG00000108001.12 | 2.25220775 | 8.18736912 | 1.48E-07 |
| ENSG00000185811.15 | 1.98292313 | 8.17595779 | 1.51E-07 |
| ENSG00000179776.16 | 1.42812476 | 8.1528146 | 1.57E-07 |
| ENSG00000121361.3 | 1.5686634 | 8.10305413 | 1.72E-07 |
| ENSG00000245812.2 | 2.08535852 | 8.23240735 | 1.36E-07 |
| ENSG00000168421.11 | 1.79048965 | 8.04092613 | 1.92E-07 |
| ENSG00000254102.1 | 3.12285078 | 8.34199252 | 1.12E-07 |
| ENSG00000138615.5 | 3.76172552 | 8.0317363 | 1.96E-07 |
| ENSG00000248491.4 | 3.29141899 | 8.19311445 | 1.46E-07 |
| ENSG00000151702.15 | 1.6289424 | 8.01093186 | 2.03E-07 |
| ENSG00000136250.10 | 1.76212573 | 7.98990629 | 2.11E-07 |
| ENSG00000143196.4 | 2.99218163 | 8.00895295 | 2.04E-07 |
| ENSG00000122224.16 | 2.35907955 | 7.96872976 | 2.19E-07 |
| ENSG00000169291.8 | 1.69729497 | 7.95810406 | 2.24E-07 |
| ENSG00000161405.15 | 2.22916116 | 7.95951714 | 2.23E-07 |
| ENSG00000137078.7 | 2.16767602 | 7.94962516 | 2.27E-07 |
| ENSG00000107317.10 | 3.30666361 | 7.99808855 | 2.08E-07 |
| ENSG00000104888.8 | 1.70200767 | 7.96167046 | 2.22E-07 |
| ENSG00000205277.8 | -1.8470164 | -7.9317415 | 2.35E-07 |
| ENSG00000124126.12 | 1.36786743 | 7.96129185 | 2.22E-07 |
| ENSG00000188404.7 | 2.55934876 | 7.91239694 | 2.43E-07 |
| ENSG00000127083.7 | 2.78581241 | 7.89892191 | 2.49E-07 |
| ENSG00000241644.2 | 2.10874337 | 7.92153958 | 2.39E-07 |
| ENSG00000267534.2 | 0.95545139 | 7.88308394 | 2.56E-07 |
| ENSG00000078589.11 | 2.14156551 | 7.88745755 | 2.54E-07 |
| ENSG00000180096.10 | 1.5539527 | 7.86180501 | 2.67E-07 |
| ENSG00000115085.12 | 1.75879282 | 7.84802688 | 2.73E-07 |
| ENSG00000117215.13 | 3.48664645 | 7.8858986 | 2.55E-07 |
| ENSG00000134533.5 | 1.64033303 | 7.84349643 | 2.76E-07 |
| ENSG00000126353.3 | 2.54887877 | 7.82983921 | 2.83E-07 |
| ENSG00000089012.13 | 1.42903802 | 7.83851119 | 2.78E-07 |
| ENSG00000119865.7 | 1.3722536 | 7.8134611 | 2.91E-07 |
| ENSG00000101445.8 | 1.93089095 | 7.79773677 | 3.00E-07 |
| ENSG00000116824.4 | 1.68679228 | 7.78407655 | 3.08E-07 |
| ENSG00000257086.1 | -1.5461716 | -7.8191512 | 2.88E-07 |
| ENSG00000162063.11 | -0.9780081 | -7.769002 | 3.16E-07 |
| ENSG00000072818.10 | 1.51625659 | 7.77385026 | 3.13E-07 |
| ENSG00000275361.1 | 2.8331942 | 7.84929105 | 2.73E-07 |
| ENSG00000064300.7 | 3.26039567 | 7.74715582 | 3.29E-07 |
| ENSG00000133561.14 | 1.94227913 | 7.73014345 | 3.40E-07 |
| ENSG00000132386.9 | 1.92822436 | 7.87979025 | 2.58E-07 |
| ENSG00000272511.1 | 2.26286927 | 7.79016083 | 3.04E-07 |
| ENSG00000237523.1 | -1.837336 | -7.7010457 | 3.59E-07 |
| ENSG00000150672.15 | 2.26696794 | 7.69197106 | 3.65E-07 |
| ENSG00000165124.16 | 2.26002716 | 7.74471935 | 3.31E-07 |
| ENSG00000132554.18 | 2.40666622 | 7.71983159 | 3.46E-07 |
| ENSG00000138356.12 | 2.74316634 | 7.6804054 | 3.73E-07 |
| ENSG00000172215.5 | 1.86490294 | 7.66814273 | 3.81E-07 |
| ENSG00000013725.13 | 1.26357001 | 7.67884 | 3.74E-07 |
| ENSG00000091986.14 | 2.72705141 | 7.84188738 | 2.77E-07 |
| ENSG00000144857.13 | 1.94760127 | 7.71391155 | 3.50E-07 |
| ENSG00000115252.17 | 1.74807123 | 7.6725666 | 3.78E-07 |
| ENSG00000245904.3 | 1.30985855 | 7.73326219 | 3.38E-07 |
| ENSG00000169413.2 | 1.69089386 | 7.66938872 | 3.80E-07 |
| ENSG00000172724.10 | 4.33522162 | 7.63891028 | 4.02E-07 |
| ENSG00000101955.13 | 2.48791375 | 7.63256763 | 4.07E-07 |
| ENSG00000224122.1 | 3.12842366 | 7.70378506 | 3.57E-07 |
| ENSG00000180353.9 | 1.60069122 | 7.65375087 | 3.91E-07 |
| ENSG00000010319.5 | 2.06281728 | 7.59128376 | 4.40E-07 |
| ENSG00000134817.10 | 2.13949784 | 7.62051025 | 4.16E-07 |
| ENSG00000161940.9 | 1.38547149 | 7.57672604 | 4.52E-07 |
| ENSG00000106948.15 | 1.18807955 | 7.61226716 | 4.23E-07 |
| ENSG00000168079.15 | 5.48577381 | 7.55459989 | 4.71E-07 |
| ENSG00000213203.2 | 1.64174228 | 7.53316462 | 4.90E-07 |
| ENSG00000101331.14 | 1.36608611 | 7.51814824 | 5.04E-07 |
| ENSG00000105122.11 | 1.27480698 | 7.52488044 | 4.98E-07 |
| ENSG00000274139.1 | -4.5987304 | -7.585251 | 4.45E-07 |
| ENSG00000144642.19 | 1.42594795 | 7.52219339 | 5.01E-07 |
| ENSG00000276557.1 | 2.28713366 | 7.65979048 | 3.87E-07 |
| ENSG00000161013.15 | -1.0824754 | -7.6116521 | 4.23E-07 |
| ENSG00000232613.5 | 2.81428661 | 7.69598546 | 3.62E-07 |
| ENSG00000179088.13 | 3.19660492 | 7.73668356 | 3.36E-07 |
| ENSG00000125810.9 | 1.65088543 | 7.57951123 | 4.50E-07 |
| ENSG00000174004.5 | 1.33430343 | 7.47422206 | 5.48E-07 |
| ENSG00000170899.9 | 1.07870101 | 7.47902905 | 5.43E-07 |
| ENSG00000248441.5 | 2.24997941 | 7.49751748 | 5.24E-07 |
| ENSG00000138378.16 | 1.68007629 | 7.46045877 | 5.62E-07 |
| ENSG00000126545.12 | 4.03787374 | 7.5114667 | 5.11E-07 |
| ENSG00000251179.1 | -1.4204116 | -7.5048578 | 5.17E-07 |
| ENSG00000166848.5 | 0.60873921 | 7.53912075 | 4.85E-07 |
| ENSG00000132837.13 | 1.65262224 | 7.46802919 | 5.54E-07 |
| ENSG00000079931.13 | 2.0802929 | 7.51625656 | 5.06E-07 |
| ENSG00000132840.8 | 1.70239078 | 7.41405708 | 6.14E-07 |
| ENSG00000164691.15 | 1.55933752 | 7.41166493 | 6.16E-07 |
| ENSG00000162998.4 | 1.7415684 | 7.44126905 | 5.83E-07 |
| ENSG00000162407.8 | 1.36879165 | 7.45419614 | 5.69E-07 |
| ENSG00000120279.6 | 1.64874693 | 7.37265473 | 6.64E-07 |
| ENSG00000244122.2 | -2.6711749 | -7.6253928 | 4.13E-07 |
| ENSG00000240687.1 | 2.29352986 | 7.46086436 | 5.62E-07 |
| ENSG00000135426.13 | 1.66906101 | 7.35387364 | 6.88E-07 |
| ENSG00000198844.9 | 1.73777509 | 7.36736641 | 6.70E-07 |
| ENSG00000164035.8 | 2.2187666 | 7.36376092 | 6.75E-07 |
| ENSG00000102245.6 | 1.87646925 | 7.37716349 | 6.58E-07 |
| ENSG00000106483.10 | 2.30071873 | 7.51195895 | 5.10E-07 |
| ENSG00000251408.1 | 3.12139889 | 7.42758698 | 5.98E-07 |
| ENSG00000147257.12 | 2.46360931 | 7.36735968 | 6.70E-07 |
| ENSG00000261269.1 | 1.48881063 | 7.3394046 | 7.07E-07 |
| ENSG00000009790.13 | 1.81680324 | 7.34106852 | 7.05E-07 |
| ENSG00000247774.5 | 1.34631831 | 7.3366488 | 7.11E-07 |
| ENSG00000225938.1 | 1.38743622 | 7.46437532 | 5.58E-07 |
| ENSG00000111269.2 | 0.71925717 | 7.41587902 | 6.12E-07 |
| ENSG00000250334.4 | 2.81235836 | 7.57634924 | 4.52E-07 |
| ENSG00000186185.12 | -1.7038343 | -7.3161031 | 7.39E-07 |
| ENSG00000278965.1 | 1.8105698 | 7.49256618 | 5.29E-07 |
| ENSG00000177098.7 | 1.87529109 | 7.3179818 | 7.36E-07 |
| ENSG00000064989.11 | 1.91254618 | 7.34446697 | 7.00E-07 |
| ENSG00000153283.11 | 1.56315506 | 7.30736148 | 7.51E-07 |
| ENSG00000114200.8 | 2.29438068 | 7.29703849 | 7.66E-07 |
| ENSG00000277734.3 | 1.67745719 | 7.32783062 | 7.23E-07 |
| ENSG00000266542.1 | -3.1570442 | -7.3645081 | 6.74E-07 |
| ENSG00000228035.1 | 3.72184588 | 7.48644899 | 5.35E-07 |
| ENSG00000176435.6 | 1.27804576 | 7.32019982 | 7.33E-07 |
| ENSG00000211753.3 | 1.67804424 | 7.25759422 | 8.26E-07 |
| ENSG00000120129.5 | 1.85910929 | 7.42625987 | 6.00E-07 |
| ENSG00000019991.14 | 1.84264887 | 7.26227112 | 8.19E-07 |
| ENSG00000095370.18 | 1.43376162 | 7.2652826 | 8.14E-07 |
| ENSG00000274172.1 | 3.55428574 | 7.34076342 | 7.05E-07 |
| ENSG00000258733.4 | 1.36519305 | 7.3071228 | 7.52E-07 |
| ENSG00000211751.6 | 1.53673936 | 7.28623778 | 7.82E-07 |
| ENSG00000126785.11 | 1.40872174 | 7.24137264 | 8.52E-07 |
| ENSG00000166265.10 | 1.80759649 | 7.23027205 | 8.71E-07 |
| ENSG00000211747.3 | 1.67992037 | 7.2351659 | 8.63E-07 |
| ENSG00000134668.11 | -2.0275678 | -7.1900913 | 9.41E-07 |
| ENSG00000126882.11 | 1.36475631 | 7.19798916 | 9.26E-07 |
| ENSG00000111796.3 | 1.75376059 | 7.14950624 | 1.02E-06 |
| ENSG00000109339.17 | 1.63859427 | 7.15628615 | 1.00E-06 |
| ENSG00000154277.11 | 1.79645286 | 7.18829376 | 9.44E-07 |
| ENSG00000171791.11 | 1.76985522 | 7.16204675 | 9.93E-07 |
| ENSG00000120075.5 | -1.6387436 | -7.1438008 | 1.03E-06 |
| ENSG00000253105.4 | 2.80763433 | 7.36495682 | 6.74E-07 |
| ENSG00000162894.10 | 2.445727 | 7.14203067 | 1.03E-06 |
| ENSG00000280265.1 | -2.9518831 | -7.33083 | 7.19E-07 |
| ENSG00000143344.14 | 1.57027698 | 7.16344043 | 9.90E-07 |
| ENSG00000091651.7 | -1.5199813 | -7.1005807 | 1.12E-06 |
| ENSG00000135953.9 | -0.8601466 | -7.1148044 | 1.09E-06 |
| ENSG00000127920.5 | 1.66841888 | 7.13742185 | 1.04E-06 |
| ENSG00000188452.12 | 1.4167678 | 7.08237962 | 1.16E-06 |
| ENSG00000196666.4 | 3.46491157 | 7.18496485 | 9.50E-07 |
| ENSG00000134259.3 | 2.10645473 | 7.08339483 | 1.16E-06 |
| ENSG00000168405.13 | 1.54567638 | 7.06359891 | 1.20E-06 |
| ENSG00000102678.6 | 1.80131644 | 7.04876694 | 1.24E-06 |
| ENSG00000108387.13 | 1.34793461 | 7.04878892 | 1.24E-06 |
| ENSG00000166851.13 | -1.6753281 | -7.0302132 | 1.28E-06 |
| ENSG00000117650.11 | -1.9323205 | -6.9855035 | 1.40E-06 |
| ENSG00000153563.14 | 2.02011968 | 7.00424553 | 1.35E-06 |
| ENSG00000238195.1 | 1.76572418 | 7.02839468 | 1.29E-06 |
| ENSG00000081237.17 | 1.98625486 | 7.04301244 | 1.25E-06 |
| ENSG00000174946.6 | 2.01833806 | 6.92414434 | 1.58E-06 |
| ENSG00000229106.1 | 3.51006069 | 6.99469538 | 1.37E-06 |
| ENSG00000147113.15 | 1.40371821 | 6.9440856 | 1.52E-06 |
| ENSG00000114279.12 | 1.61613026 | 6.89436416 | 1.67E-06 |
| ENSG00000277301.1 | 2.66691498 | 7.07193432 | 1.18E-06 |
| ENSG00000197992.5 | 2.16226147 | 6.92098301 | 1.59E-06 |
| ENSG00000250048.1 | 3.51849606 | 6.90543389 | 1.63E-06 |
| ENSG00000066056.12 | 1.21951653 | 6.92899828 | 1.56E-06 |
| ENSG00000145147.18 | 2.3159805 | 6.91988271 | 1.59E-06 |
| ENSG00000110324.8 | 1.55843707 | 6.92694318 | 1.57E-06 |
| ENSG00000162892.14 | 2.29415771 | 6.85590578 | 1.80E-06 |
| ENSG00000172260.12 | 2.12734515 | 6.89015976 | 1.68E-06 |
| ENSG00000153233.11 | -2.3839836 | -6.8534077 | 1.81E-06 |
| ENSG00000181847.10 | 1.76034246 | 6.85240737 | 1.81E-06 |
| ENSG00000135919.11 | 2.3416145 | 6.93689599 | 1.54E-06 |
| ENSG00000255987.1 | 1.656964 | 6.98049885 | 1.41E-06 |
| ENSG00000121067.16 | 0.55493819 | 6.91968073 | 1.59E-06 |
| ENSG00000091831.20 | 2.1081174 | 6.82694428 | 1.91E-06 |
| ENSG00000174804.3 | 1.7676044 | 6.88691074 | 1.70E-06 |
| ENSG00000279036.1 | 3.92346046 | 6.90547333 | 1.63E-06 |
| ENSG00000139549.2 | 1.90919926 | 6.86342785 | 1.78E-06 |
| ENSG00000123159.14 | -1.4862012 | -6.9515913 | 1.49E-06 |
| ENSG00000211899.6 | 3.23436095 | 6.98225258 | 1.41E-06 |
| ENSG00000231768.1 | 1.70103567 | 6.84358128 | 1.85E-06 |
| ENSG00000245164.5 | 2.64906769 | 6.7928197 | 2.04E-06 |
| ENSG00000011465.15 | 1.99418853 | 7.07186782 | 1.18E-06 |
| ENSG00000163909.7 | 1.67320727 | 6.87608466 | 1.73E-06 |
| ENSG00000259834.1 | 2.35578376 | 6.77948573 | 2.09E-06 |
| ENSG00000180304.13 | 0.59987498 | 6.88112227 | 1.71E-06 |
| ENSG00000078900.13 | -2.0598256 | -6.7742045 | 2.12E-06 |
| ENSG00000143867.6 | 3.01675681 | 6.76738039 | 2.15E-06 |
| ENSG00000148053.14 | 3.16185819 | 6.80775803 | 1.98E-06 |
| ENSG00000075702.15 | -1.4847872 | -6.7619598 | 2.17E-06 |
| ENSG00000174348.12 | 1.68722132 | 6.88986332 | 1.69E-06 |
| ENSG00000228401.4 | 1.83258376 | 6.85104419 | 1.82E-06 |
| ENSG00000185565.10 | 1.61153869 | 6.81838771 | 1.94E-06 |
| ENSG00000275301.1 | 3.30648766 | 6.80004639 | 2.01E-06 |
| ENSG00000228789.5 | 3.12899145 | 6.80679785 | 1.98E-06 |
| ENSG00000115380.17 | 2.45721585 | 6.88978033 | 1.69E-06 |
| ENSG00000187741.13 | -1.3705097 | -6.751479 | 2.21E-06 |
| ENSG00000160856.19 | 3.28780013 | 6.73441478 | 2.29E-06 |
| ENSG00000128815.16 | 1.86090592 | 6.7606233 | 2.17E-06 |
| ENSG00000113263.11 | 2.15333834 | 6.72740067 | 2.32E-06 |
| ENSG00000122420.8 | 2.28505995 | 6.71282042 | 2.39E-06 |
| ENSG00000109472.12 | 2.43194466 | 6.8780443 | 1.72E-06 |
| ENSG00000101082.12 | 1.39588183 | 6.70555203 | 2.43E-06 |
| ENSG00000275302.1 | 1.68276996 | 6.70313011 | 2.44E-06 |
| ENSG00000010810.16 | 1.0871901 | 6.78855779 | 2.06E-06 |
| ENSG00000080546.12 | 1.24542398 | 6.74949889 | 2.22E-06 |
| ENSG00000147862.13 | 1.33024032 | 6.78989863 | 2.05E-06 |
| ENSG00000081189.12 | 1.43237617 | 6.74538372 | 2.24E-06 |
| ENSG00000112964.12 | 2.36001964 | 6.69133378 | 2.49E-06 |
| ENSG00000173578.7 | 2.43348036 | 6.6802477 | 2.55E-06 |
| ENSG00000077942.16 | 2.22293542 | 6.90837631 | 1.63E-06 |
| ENSG00000204622.9 | -1.8610169 | -6.6705403 | 2.60E-06 |
| ENSG00000156298.11 | 2.66744307 | 6.70204463 | 2.44E-06 |
| ENSG00000166562.7 | 1.2372046 | 6.74379674 | 2.25E-06 |
| ENSG00000105639.17 | 1.14715721 | 6.72203115 | 2.35E-06 |
| ENSG00000111913.14 | 2.3027944 | 6.67241994 | 2.59E-06 |
| ENSG00000267414.1 | 1.64856304 | 6.65842201 | 2.66E-06 |
| ENSG00000180772.6 | 5.07191431 | 6.66843894 | 2.61E-06 |
| ENSG00000154175.15 | 2.37862619 | 6.72212898 | 2.35E-06 |
| ENSG00000172349.15 | 1.79359428 | 6.69604366 | 2.47E-06 |
| ENSG00000122122.9 | 1.59600919 | 6.68930601 | 2.51E-06 |
| ENSG00000115956.9 | 1.37707682 | 6.68108886 | 2.55E-06 |
| ENSG00000213088.8 | 3.34531995 | 6.68666479 | 2.52E-06 |
| ENSG00000165028.10 | 0.76970313 | 6.62369556 | 2.86E-06 |
| ENSG00000177432.6 | 1.39023831 | 6.61856423 | 2.88E-06 |
| ENSG00000274961.1 | 3.07642144 | 6.64835661 | 2.72E-06 |
| ENSG00000127564.15 | -2.2267489 | -6.6050552 | 2.96E-06 |
| ENSG00000111077.16 | 1.16568866 | 6.73147952 | 2.30E-06 |
| ENSG00000234460.1 | -3.4229695 | -6.6275993 | 2.83E-06 |
| ENSG00000270318.1 | 3.3276526 | 6.65419331 | 2.69E-06 |
| ENSG00000198471.1 | -3.1654428 | -6.6247074 | 2.85E-06 |
| ENSG00000157224.14 | -1.0615355 | -6.6927602 | 2.49E-06 |
| ENSG00000112335.13 | 0.53704943 | 6.73812955 | 2.27E-06 |
| ENSG00000133083.13 | 2.14447876 | 6.61656704 | 2.90E-06 |
| ENSG00000174123.9 | 2.63176865 | 6.58587216 | 3.08E-06 |
| ENSG00000173198.5 | 1.8285443 | 6.5800818 | 3.12E-06 |
| ENSG00000225096.1 | 2.23213882 | 6.64770866 | 2.72E-06 |
| ENSG00000122188.11 | 2.40675699 | 6.57489539 | 3.15E-06 |
| ENSG00000134516.14 | 1.41465629 | 6.64735826 | 2.72E-06 |
| ENSG00000129675.14 | 1.3732249 | 6.63396621 | 2.80E-06 |
| ENSG00000226571.1 | 3.14262786 | 6.66037923 | 2.65E-06 |
| ENSG00000106952.6 | 1.88214385 | 6.56733003 | 3.20E-06 |
| ENSG00000005844.16 | 1.50578445 | 6.62424384 | 2.85E-06 |
| ENSG00000141736.12 | -1.1425982 | -6.7438584 | 2.25E-06 |
| ENSG00000145649.7 | 1.84006465 | 6.56733678 | 3.20E-06 |
| ENSG00000255471.1 | 1.84789373 | 6.57957445 | 3.12E-06 |
| ENSG00000267097.1 | 2.884686 | 6.67881887 | 2.56E-06 |
| ENSG00000163239.11 | 1.79355591 | 6.54548987 | 3.34E-06 |
| ENSG00000183813.6 | 2.28375206 | 6.53693089 | 3.40E-06 |
| ENSG00000248307.4 | 4.0725408 | 6.62946785 | 2.82E-06 |
| ENSG00000145362.15 | 2.4754574 | 6.57694089 | 3.14E-06 |
| ENSG00000211724.3 | 1.78808452 | 6.63188816 | 2.81E-06 |
| ENSG00000196364.9 | -2.7725 | -6.5544384 | 3.28E-06 |
| ENSG00000124785.7 | 1.93269885 | 6.51636193 | 3.54E-06 |
| ENSG00000183160.8 | 1.99238119 | 6.62366264 | 2.86E-06 |
| ENSG00000232973.10 | 1.53355291 | 6.49456739 | 3.70E-06 |
| ENSG00000175063.15 | -1.9901971 | -6.5187443 | 3.52E-06 |
| ENSG00000102181.18 | 1.00734328 | 6.59404661 | 3.03E-06 |
| ENSG00000150636.14 | 1.70488812 | 6.49729502 | 3.68E-06 |
| ENSG00000246662.5 | 1.57672272 | 6.54934534 | 3.31E-06 |
| ENSG00000263482.3 | 2.49320336 | 6.60657821 | 2.95E-06 |
| ENSG00000228016.1 | 2.73534186 | 6.50350946 | 3.63E-06 |
| ENSG00000147100.8 | 0.93926247 | 6.53845923 | 3.39E-06 |
| ENSG00000233197.1 | -1.6375441 | -6.5001119 | 3.66E-06 |
| ENSG00000246082.2 | -0.9555316 | -6.4751063 | 3.85E-06 |
| ENSG00000139567.11 | 1.03211642 | 6.55123678 | 3.30E-06 |
| ENSG00000258572.1 | 2.32696313 | 6.54137542 | 3.37E-06 |
| ENSG00000249242.6 | 1.59951884 | 6.48620976 | 3.76E-06 |
| ENSG00000241106.5 | 1.841989 | 6.46107796 | 3.96E-06 |
| ENSG00000084070.10 | 1.29263791 | 6.58062313 | 3.11E-06 |
| ENSG00000134201.9 | 1.83121807 | 6.47589205 | 3.84E-06 |
| ENSG00000268080.2 | 2.73827706 | 6.47638425 | 3.84E-06 |
| ENSG00000053702.13 | 1.07180434 | 6.45385176 | 4.02E-06 |
| ENSG00000279204.1 | 2.02501641 | 6.45780309 | 3.99E-06 |
| ENSG00000197291.7 | 1.72123484 | 6.44522037 | 4.09E-06 |
| ENSG00000105246.5 | 1.64100527 | 6.43162896 | 4.20E-06 |
| ENSG00000227308.2 | -2.831343 | -6.4724079 | 3.87E-06 |
| ENSG00000163565.17 | 0.89761996 | 6.58415315 | 3.09E-06 |
| ENSG00000273238.1 | 2.32322109 | 6.55989431 | 3.24E-06 |
| ENSG00000110934.9 | 1.13645735 | 6.45099892 | 4.04E-06 |
| ENSG00000101938.13 | 3.93467478 | 6.46761593 | 3.91E-06 |
| ENSG00000196569.10 | 1.72622145 | 6.53636326 | 3.40E-06 |
| ENSG00000067798.12 | 1.78095584 | 6.43282725 | 4.19E-06 |
| ENSG00000134690.9 | -1.6096943 | -6.4274338 | 4.24E-06 |
| ENSG00000180440.3 | 3.83816868 | 6.43102504 | 4.21E-06 |
| ENSG00000265107.2 | 1.49838855 | 6.43661888 | 4.16E-06 |
| ENSG00000113296.13 | 2.82761959 | 6.53853275 | 3.39E-06 |
| ENSG00000171951.4 | 2.96113153 | 6.50029784 | 3.66E-06 |
| ENSG00000250387.2 | 3.62827564 | 6.41889827 | 4.31E-06 |
| ENSG00000260293.2 | 1.53614738 | 6.40747961 | 4.41E-06 |
| ENSG00000151468.10 | 1.64895305 | 6.45628121 | 4.00E-06 |
| ENSG00000121966.6 | 1.6760734 | 6.5331124 | 3.42E-06 |
| ENSG00000160113.5 | -1.2355283 | -6.4781221 | 3.82E-06 |
| ENSG00000099260.9 | 1.84837224 | 6.41584016 | 4.34E-06 |
| ENSG00000270071.1 | 1.30827281 | 6.37811668 | 4.68E-06 |
| ENSG00000130300.7 | 1.19616099 | 6.54784646 | 3.32E-06 |
| ENSG00000129993.13 | 1.36682365 | 6.38066933 | 4.66E-06 |
| ENSG00000253364.1 | 4.73547559 | 6.41844841 | 4.32E-06 |
| ENSG00000129173.11 | -1.5539397 | -6.3609806 | 4.85E-06 |
| ENSG00000171115.3 | 1.82005011 | 6.39976269 | 4.48E-06 |
| ENSG00000162618.11 | 1.45857501 | 6.41911422 | 4.31E-06 |
| ENSG00000118523.5 | 2.15862818 | 6.60218247 | 2.98E-06 |
| ENSG00000261472.1 | 3.04552109 | 6.37186201 | 4.74E-06 |
| ENSG00000186212.3 | -1.9131055 | -6.3621888 | 4.84E-06 |
| ENSG00000079805.15 | -1.1275696 | -6.5420602 | 3.36E-06 |
| ENSG00000233485.1 | -1.8483131 | -6.3810255 | 4.66E-06 |
| ENSG00000130037.4 | 2.72356297 | 6.34005889 | 5.06E-06 |
| ENSG00000187109.12 | 0.77631503 | 6.55496439 | 3.28E-06 |
| ENSG00000140285.8 | 2.32031153 | 6.37638341 | 4.70E-06 |
| ENSG00000211911.1 | 1.84000897 | 6.33563505 | 5.11E-06 |
| ENSG00000106991.12 | 1.11729349 | 6.49319739 | 3.71E-06 |
| ENSG00000228742.8 | -4.6934309 | -6.3114088 | 5.36E-06 |
| ENSG00000204176.12 | 1.73791939 | 6.34322613 | 5.03E-06 |
| ENSG00000268001.1 | 1.0674039 | 6.29725351 | 5.52E-06 |
| ENSG00000166501.11 | 2.02165337 | 6.32936585 | 5.17E-06 |
| ENSG00000228944.1 | 2.20437672 | 6.31778077 | 5.30E-06 |
| ENSG00000244681.1 | 3.10720062 | 6.38741076 | 4.60E-06 |
| ENSG00000123572.15 | 2.48262787 | 6.30095343 | 5.48E-06 |
| ENSG00000085552.15 | -2.7376996 | -6.3224276 | 5.25E-06 |
| ENSG00000152583.11 | 2.13233679 | 6.49192674 | 3.72E-06 |
| ENSG00000237513.1 | 1.58975553 | 6.28485885 | 5.66E-06 |
| ENSG00000196329.9 | 1.89182034 | 6.26631119 | 5.88E-06 |
| ENSG00000163508.11 | 1.99813459 | 6.25846465 | 5.98E-06 |
| ENSG00000213424.7 | 2.86649093 | 6.27355836 | 5.80E-06 |
| ENSG00000125245.11 | 2.32759485 | 6.25442246 | 6.03E-06 |
| ENSG00000164985.13 | 1.09257564 | 6.34837276 | 4.98E-06 |
| ENSG00000266312.1 | 2.23718124 | 6.30882605 | 5.39E-06 |
| ENSG00000124507.9 | 1.82815284 | 6.23491579 | 6.27E-06 |
| ENSG00000279673.1 | -1.1612051 | -6.2348546 | 6.27E-06 |
| ENSG00000211445.10 | 2.41470678 | 6.38121877 | 4.65E-06 |
| ENSG00000013297.9 | 1.69816363 | 6.282083 | 5.70E-06 |
| ENSG00000163644.13 | 1.45150194 | 6.28906812 | 5.62E-06 |
| ENSG00000239857.5 | -1.1514867 | -6.2218838 | 6.44E-06 |
| ENSG00000119888.9 | -1.5053198 | -6.4342491 | 4.18E-06 |
| ENSG00000154734.13 | 2.40204858 | 6.33765372 | 5.09E-06 |
| ENSG00000100351.15 | 1.42252374 | 6.21809516 | 6.49E-06 |
| ENSG00000099282.8 | -1.6596787 | -6.3523941 | 4.93E-06 |
| ENSG00000111665.10 | -1.2994136 | -6.2219121 | 6.44E-06 |
| ENSG00000251573.2 | 3.05033159 | 6.25871391 | 5.97E-06 |
| ENSG00000255585.3 | -2.0746897 | -6.1987864 | 6.76E-06 |
| ENSG00000179528.14 | -1.9048478 | -6.1961107 | 6.79E-06 |
| ENSG00000123485.10 | -1.5609262 | -6.2123865 | 6.57E-06 |
| ENSG00000272763.1 | -3.4469661 | -6.1913829 | 6.86E-06 |
| ENSG00000158402.17 | -1.8592384 | -6.1897033 | 6.88E-06 |
| ENSG00000152092.14 | 2.72433599 | 6.18978453 | 6.88E-06 |
| ENSG00000134853.10 | 1.79050908 | 6.34509464 | 5.01E-06 |
| ENSG00000167216.15 | 1.01698471 | 6.18639854 | 6.93E-06 |
| ENSG00000121064.11 | 0.96903467 | 6.31673355 | 5.31E-06 |
| ENSG00000185610.6 | 2.75324558 | 6.26546203 | 5.89E-06 |
| ENSG00000133104.11 | 1.17096953 | 6.27273336 | 5.81E-06 |
| ENSG00000006555.9 | -1.427733 | -6.2368874 | 6.25E-06 |
| ENSG00000197245.4 | 1.75350173 | 6.16806775 | 7.20E-06 |
| ENSG00000266524.2 | 1.79598317 | 6.17162265 | 7.14E-06 |
| ENSG00000160219.10 | 1.04090588 | 6.18358754 | 6.97E-06 |
| ENSG00000139182.12 | 0.94891279 | 6.2919384 | 5.58E-06 |
| ENSG00000267107.5 | 1.39275217 | 6.16604335 | 7.23E-06 |
| ENSG00000176194.16 | 6.24474084 | 6.15818816 | 7.35E-06 |
| ENSG00000069431.9 | 2.17563393 | 6.20740323 | 6.64E-06 |
| ENSG00000160957.11 | -1.415455 | -6.1729925 | 7.12E-06 |
| ENSG00000170275.13 | 0.71839372 | 6.33808083 | 5.08E-06 |
| ENSG00000155961.4 | 2.02328857 | 6.13789467 | 7.66E-06 |
| ENSG00000176160.8 | 2.30364097 | 6.22533745 | 6.40E-06 |
| ENSG00000010327.9 | 1.58475658 | 6.27623322 | 5.76E-06 |
| ENSG00000173757.8 | 0.69628155 | 6.2711795 | 5.82E-06 |
| ENSG00000233293.1 | 3.53797325 | 6.2074626 | 6.64E-06 |
| ENSG00000234595.1 | 3.04077541 | 6.14819265 | 7.50E-06 |
| ENSG00000231943.6 | 2.8796701 | 6.20822354 | 6.63E-06 |
| ENSG00000280182.1 | -2.2171531 | -6.1841251 | 6.96E-06 |
| ENSG00000007312.11 | 3.08486713 | 6.1398251 | 7.63E-06 |
| ENSG00000104490.16 | 1.40915398 | 6.19454028 | 6.82E-06 |
| ENSG00000178623.10 | -2.1915694 | -6.1850621 | 6.95E-06 |
| ENSG00000204065.2 | 2.51503322 | 6.11421726 | 8.04E-06 |
| ENSG00000113532.11 | 1.42004808 | 6.16840584 | 7.19E-06 |
| ENSG00000196878.11 | -1.9664628 | -6.361517 | 4.84E-06 |
| ENSG00000261373.1 | -1.6552274 | -6.1103847 | 8.11E-06 |
| ENSG00000211923.1 | 1.71773524 | 6.11720293 | 7.99E-06 |
| ENSG00000107758.14 | 0.65732258 | 6.22375078 | 6.42E-06 |
| ENSG00000138111.13 | -1.0813282 | -6.1347833 | 7.71E-06 |
| ENSG00000226871.1 | 2.70147799 | 6.1370444 | 7.67E-06 |
| ENSG00000178878.11 | 2.15242868 | 6.19997317 | 6.74E-06 |
| ENSG00000189058.7 | 2.32484515 | 6.29933258 | 5.50E-06 |
| ENSG00000136297.13 | 1.93596256 | 6.11141166 | 8.09E-06 |
| ENSG00000225649.4 | 2.24853542 | 6.12706228 | 7.83E-06 |
| ENSG00000134640.2 | 2.56428227 | 6.10990909 | 8.11E-06 |
| ENSG00000139970.15 | 2.28727825 | 6.13874134 | 7.65E-06 |
| ENSG00000160877.5 | -0.7255224 | -6.2112745 | 6.59E-06 |
| ENSG00000250957.1 | 2.72402785 | 6.1600359 | 7.32E-06 |
| ENSG00000149488.12 | 2.46668177 | 6.12314838 | 7.90E-06 |
| ENSG00000165424.6 | 1.33208608 | 6.20757929 | 6.64E-06 |
| ENSG00000145708.9 | 2.57087687 | 6.1007755 | 8.27E-06 |
| ENSG00000167513.7 | -1.8338956 | -6.0786333 | 8.66E-06 |
| ENSG00000087586.16 | -1.2523872 | -6.0782838 | 8.66E-06 |
| ENSG00000160654.8 | 1.77889066 | 6.05950067 | 9.01E-06 |
| ENSG00000160408.13 | 0.91765959 | 6.18212975 | 6.99E-06 |
| ENSG00000261448.1 | 1.44291932 | 6.07837176 | 8.66E-06 |
| ENSG00000163053.9 | 1.0940369 | 6.06366451 | 8.93E-06 |
| ENSG00000205221.11 | 2.83595821 | 6.03981869 | 9.38E-06 |
| ENSG00000281406.1 | -2.8847959 | -6.0516029 | 9.16E-06 |
| ENSG00000164483.15 | 1.66149862 | 6.03421409 | 9.49E-06 |
| ENSG00000141753.6 | 1.51484475 | 6.35086907 | 4.95E-06 |
| ENSG00000276575.1 | -1.8337713 | -6.0469646 | 9.24E-06 |
| ENSG00000226979.7 | 1.82777623 | 6.02860796 | 9.60E-06 |
| ENSG00000228917.1 | -2.7980384 | -6.0836171 | 8.57E-06 |
| ENSG00000196338.11 | 1.31076477 | 6.03979281 | 9.38E-06 |
| ENSG00000163827.11 | 2.10005968 | 6.0181178 | 9.82E-06 |
| ENSG00000226660.2 | 2.12952822 | 6.06071982 | 8.98E-06 |
| ENSG00000123338.11 | 1.36106862 | 6.12351912 | 7.89E-06 |
| ENSG00000162337.10 | -1.0696666 | -6.1802135 | 7.02E-06 |
| ENSG00000176532.3 | -1.9677588 | -6.081802 | 8.60E-06 |
| ENSG00000267073.1 | -3.297893 | -6.0668674 | 8.87E-06 |
| ENSG00000161888.10 | -1.6820836 | -6.0204222 | 9.77E-06 |
| ENSG00000251258.1 | 3.02921658 | 6.0239372 | 9.70E-06 |
| ENSG00000267801.1 | -1.6358303 | -6.0013088 | 1.02E-05 |
| ENSG00000123329.16 | 1.21440676 | 6.06543203 | 8.90E-06 |
| ENSG00000112137.15 | 1.07727117 | 6.03541255 | 9.47E-06 |
| ENSG00000130595.15 | 3.77691769 | 6.00371361 | 1.01E-05 |
| ENSG00000161649.11 | 4.6026181 | 6.02794679 | 9.62E-06 |
| ENSG00000163191.5 | -1.2156186 | -6.2886599 | 5.62E-06 |
| ENSG00000171587.13 | 2.4963159 | 5.99362429 | 1.03E-05 |
| ENSG00000251169.2 | -2.2664035 | -5.9874253 | 1.05E-05 |
| ENSG00000186105.7 | 1.92756782 | 5.99139445 | 1.04E-05 |
| ENSG00000014216.14 | -0.7648217 | -6.2099304 | 6.60E-06 |
| ENSG00000272282.1 | 1.64638879 | 5.98076423 | 1.06E-05 |
| ENSG00000175857.7 | 1.94703678 | 5.98418226 | 1.05E-05 |
